# Capnography monitoring reduces incidence of hypoxia in older patients undergoing gastrointestinal endoscopy under propofol sedation

**DOI:** 10.1055/a-2663-6372

**Published:** 2025-08-07

**Authors:** Qiuyue Lian, Jianbo Wu, Jie Zhang, Yizhe Zhang, Xiangyang Cheng, Xiaomei Yang, Renlong Zhou, Yue Chen, Weiwei Ding, Guangzhi Wang, Weifeng Yu, Jiaqiang Zhang, Diansan Su

**Affiliations:** 1 71140 Anesthesiology, Shanghai Jiao Tong University School of Medicine Affiliated Renji Hospital, Shanghai, China; 2 66310 Anesthesiology and Perioperative Medicine, The First Affiliated Hospital of Shandong First Medical University, Jinan, China; 3 71069 Anesthesiology, The First Affiliated Hospital of Hospital of Zhejiang University, School of Medicine, Hangzhou, China; 4 91623 Anesthesiology, Qilu Hospital of Shandong University, Jinan, China; 5 89632 Anesthesiology and Perioperative Medicine, Henan Provincial People's Hospital, Zhengzhou, China

**Keywords:** Quality and logistical aspects, Sedation and monitoring, Quality management, Performance and complications

## Abstract

**Background and study aims:**

Whether routine capnography monitoring during gastrointestinal endoscopy sedation can reduce occurrence of hypoxia is controversial. Older patients are more prone to hypoxia. This study aimed to determine the effect of additional capnography monitoring on incidence of hypoxia in older patients undergoing gastrointestinal endoscopy under propofol sedation.

**Patients and methods:**

A multicenter, randomized, single-blind, two-arm, parallel-group, controlled with an active comparator, interventional superiority clinical trial was performed at three teaching hospitals in China between September 1, 2021, and September 1, 2022. This study compared additional capnography monitoring (intervention group) and standard monitoring (control group) among older patients (aged 65–79 years) undergoing gastrointestinal endoscopy under propofol sedation. The primary outcome was incidence of hypoxia (75%-89% for < 60s). Secondary outcomes were incidence of subclinical hypoxia (90%-94%), incidence of severe hypoxia (< 75% for any duration or 75%-89% for ≥ 60s), and other adverse events (AEs).

**Results:**

Data from 1777 participants (888 intervention, 889 control group) were analyzed. Additional capnography monitoring reduced incidence of hypoxia in older patients from 19% to 12% ( *P* < 0.001). Incidence of subclinical hypoxia in the additional capnographymonitoring group was 23% and in the standard monitoring group was 15% ( *P* < 0.001). There was no significant difference in incidence of severe hypoxia ( *P* = 0.070) and other AEs between the two groups ( *P* = 0.374).

**Conclusions:**

Additional capnography monitoring during gastrointestinal endoscopy for older patients who were sedated with propofol reduces incidence of hypoxia.

## Introduction

In China, sedation is administered to 48% and 49% of patients undergoing gastroscopy and colonoscopy, and the overall number of gastrointestinal endoscopies could reach 51 million by 2030 based on the growth rate of aging 1 . Over 90% of patients undergoing gastrointestinal endoscopy in Europe and North America receive sedation 2 3 . 1

Although propofol is a better sedative drug than midazolam 4 , hypoxia is a common adverse event (AE) that occurs during gastrointestinal endoscopy under propofol sedation, especially in older patients 5 6 . Severe hypoxia can cause myocardial ischemia, cardiac arrhythmia, permanent neurologic damage, or even death 7 . Finger pulse oxygen (SpO2) is one of the routine clinical monitoring indicators in sedated gastrointestinal endoscopy 8 . However, the SpO2 value does not accurately reflect real-time ventilation of patients 9 . Studies have reported a time difference of up to 2 minutes between altered breathing patterns and hypoxia 10 11 . Incidence of and mortality from sedation-related complications can be reduced if effective interventions can be given within this time difference. As a more sensitive near-real-time monitoring index, capnography monitoring can detect hypoventilation and allow time to correct it and avoid hypoxia 12 13 . However, whether capnography monitoring should be a routine clinical indicator for monitoring in sedation gastrointestinal endoscopy is controversial 14 15 16 .

Capnography monitoring has been reported to reduce incidence of hypoxia in patients undergoing gastroscopy and colonoscopy under propofol sedation and improves patient safety during procedural sedation for elective endoscopic retrograde cholangiopancreatography (ERCP)/endoscopic ultrasonography (EUS) 17 18 19 20 . Studies have shown that additional capnography monitoring of sedated ERCP did not lower incidence of hypoxia 21 . According to international guidelines, capnography monitoring can reduce the number of episodes of apnea and hypoxia during long-duration endoscopy. Notwithstanding, it is currently not recommended as standard monitoring during endoscopy 15 16 .

Older patients may suffer from various comorbidities, which increase morbidity and mortality in the perioperative period 22 23 24 25 . In addition, older patients have a reduced ventilatory response to hypoxia and hypercapnia and are more likely to experience hypoxia during examinations that involved of application of sedative drugs, such as sedated gastrointestinal endoscopy 25 . Therefore, this study aimed to explore whether monitoring end-tidal carbon dioxide concentration in older patients undergoing sedated gastrointestinal endoscopy can effectively reduce incidence of hypoxia.

## Patients and methods

### Study design

This was a multicenter, randomized, single-blind, two-arm, parallel-group, controlled with an active comparator, interventional superiority clinical trial conducted between September 1, 2021, and September 1, 2022, in three teaching hospitals: Renji Hospital, Shanghai Jiaotong University School of Medicine, Henan Provincial People's Hospital, and Qilu Hospital of ShanDong University. The trial protocol has been published in the journal Trials 26 . This trial was approved by the Ethics Committee of Renji Hospital, Shanghai Jiao Tong University School of Medicine (approval NO. KY2021–014) and registered with ClinicalTrials.gov (NCT05030870). All participants provided informed consent.

### Participants

Patients aged 65 to 79 years who visited the three centers for inpatient and outpatient gastroscopy, colonoscopy, or gastroscopy combined with colonoscopy examination were enrolled, but those undergoing advanced upper and lower endoscopy and ERCP/EUS were not enrolled. Inclusion and exclusion criteria were evaluated by means of in-person interviews and medical record review. Inclusion criteria were patients aged 65 to 79 years, those scheduled to undergo gastrointestinal endoscopy under sedation, those who could provide informed consent, and those with American Society of Anesthesiologists (ASA) classification I-II. Exclusion criteria were existing coagulation disorders or a tendency for nose bleeding, existing episodes/ exacerbation of congestive heart failure that required a change in medication, diet, or hospitalization from any cause in the last 6 months, existing severe aortic stenosis or mitral stenosis, cardiac surgery involving thoracotomy (e.g., coronary artery bypass graft and valve replacement surgery) in the last 6 months, acute myocardial infarction in the last 6 months, existing bradycardia (heart rate < 50 bpm) or hypoxia (SpO _2_ < 90%), needing supplemental oxygen because of preexisting diseases, existing multiple trauma, existing upper respiratory tract infection, allergy to propofol or tape and adhesives, and unwillingness to comply with the protocol or procedures.

### Trial design

Patients were randomly assigned to two groups: the capnography monitoring group (the intervention group) and the standard monitoring group (the control group). Routine monitoring of heart rate, SpO _2_ , electrocardiogram, and noninvasive blood pressure (measured every 3 min) was performed before induction of anesthesia. A nasal oxygen cannula with a sampling port for collecting carbon dioxide exhaled through the mouth and nose was used (Capnostream 20, Medtronic, Inc.). Oxygen was provided at a rate of 2 L/min.

In the intervention group, the port of the nasal oxygen cannula was connected to a capnography monitoring device (Capnostream 20, Medtronic, Inc.). This device can display waveforms and values of exhaled carbon dioxide. In the control group, the nasal oxygen cannula was not connected to the capnography monitoring device, so only the SpO _2_ value could be read.

Both groups received intravenous (IV) propofol (1–2 mg/kg) and sufentanil (5–7.5 µg) boluses for anesthesia induction for gastroscopy alone and colonoscopy alone, we gave patients sufentanil 5 µg. For gastroscopy combined with colonoscopy, we gave the patients sufentanil 7.5 µg. The dosage of sufentanil was not adjusted by body weight. After reaching a moderate-to-deep sedation level by an anesthesiologist (Observer’s Assessment of Alertness/Sedation [OAA/S]) score of 2 or 3 27 , gastroscopy, colonoscopy, or gastroscopy combined with colonoscopy was initiated. During the procedure, propofol was titrated at 0.2 to 0.5 mg/kg each time, ensuring OAA/S ≤ 3 until the end of the procedure. The dosage of sufentanil was no longer added.

In both groups, interventions were performed if any sign of inadequate alveolar ventilation was observed during IV anesthesia. Interventions include increasing oxygen flow, jaw thrust, placement of a nasopharyngeal airway and jaw bracing, manual mask positive pressure ventilation, and ventilator-assisted ventilation with tracheal intubation. Alveolar hyperventilation refers to altered ventilation, apnea, and/or decreased oxygen saturation.

For the intervention group, if the expired carbon dioxide wave height decreased by half or more from baseline, positive intraoperative ventilation change was recorded; if the carbon dioxide waveform disappeared, positive intraoperative apnea was recorded; if SpO _2_ < 90%, positive intraoperative hypoxia was recorded. For the control group, if intraoperative SpO _2_ < 90%, positive intraoperative hypoxia was recorded. If either of the above occurred, the airway was opened in sequence until SpO _2_ ≥ 90%, and the last means of airway opening was recorded. In this trial, absence of exhaled CO _2_ was the capnography criterion for apnea. Altered respiration was defined as reduction in end-tidal CO _2_ of more than half of baseline, as shown by the capnography monitoring.

### Outcomes

The primary study outcome was incidence of hypoxia in older patients during sedation, defined as 75% to 89% for < 60s. Sedation period was defined as the period from start of the first medication administration to disconnection of electronic monitoring after completion of the procedure.

Secondary outcomes were incidence of subclinical respiratory depression (90%-94%) in both groups from the beginning to the end of the procedure, incidence of severe hypoxia (< 75% for any duration or 75%-89% for ≥ 60s) in both groups from the beginning to the end of the procedure, and incidence of other AEs recorded by tools proposed by the World Society of Intravenous Anesthesia International Sedation Task Force 28 .

Monitored variables and AEs were collected by observers during the procedure.

### Randomization and sample size estimation

The participants were allocated using a central randomization system mini program for each study site. In this trial, stratified blocked randomization was used to design the central randomization system. Participants were randomly assigned to the control or intervention groups in a 1:1 ratio based on the allocation sequence of the central randomization system. Length of a random sequence was not fixed, and 4, 6, and 8 were random. Only the anesthetists had access to randomized group results, and the endoscopy team did not participate in the randomization allocation process. Similarly, participants were not informed of the specific results of the allocation.

Sample size was calculated using PASS 11.0 statistical software. In our previous study, incidence of hypoxia in patients during gastrointestinal endoscopy under propofol sedation was approximately 8% 7 (oxygen was provided at a rate of 2 L/min). According to our previous clinical experience, we assumed that the intervention group reduced incidence of hypoxia from 8% to 4%, which means that the anticipated effect size of additional capnography monitoring was 50%. Results of the conventional analysis were compared between the intervention and control groups to detect the difference in proportions (hypoxia). Sample size was calculated assuming a 1:1 randomization, a power of 1 − *β* of 0.90, a two-sided α level of 5%, and a dropout rate of 10%. Thus, the required sample size was determined to be 900 in each group.

### Statistical analysis

All statistical analyses were performed using SPSS software version 26 (IBM SPSS Statistics, Armonk, New York, United States) 29 . A two-tailed *P* < 0.05 was considered statistically significant. The dropout rate was compared between groups using the chi-square or Fisher's exact probability test. The reasons for dropout were described in detail. We used the Kolmogorov-Smirnov tests for normality analysis. Baseline characteristics were presented as mean (standard deviation) for normally distributed continuous variables, median (interquartile range [IQR]) for non-normal distributed continuous variables and frequency (%) for categorical variables. Differences in baseline characteristics were assessed using unpaired *t* -tests or Mann–Whitney U tests for continuous variables and chi-square tests for categorical variables. Primary and secondary outcomes including incidence of hypoxia, subclinical respiratory depression, and severe hypoxia in the two groups were compared by chi-square tests. A mixed-effects model was used to fit repeated measurement data. AEs were compared between the two groups using the chi-square or Fisher's exact probability test. AEs and adverse reactions were expressed as frequency and percentage.

## Results

**Fig. 1:**
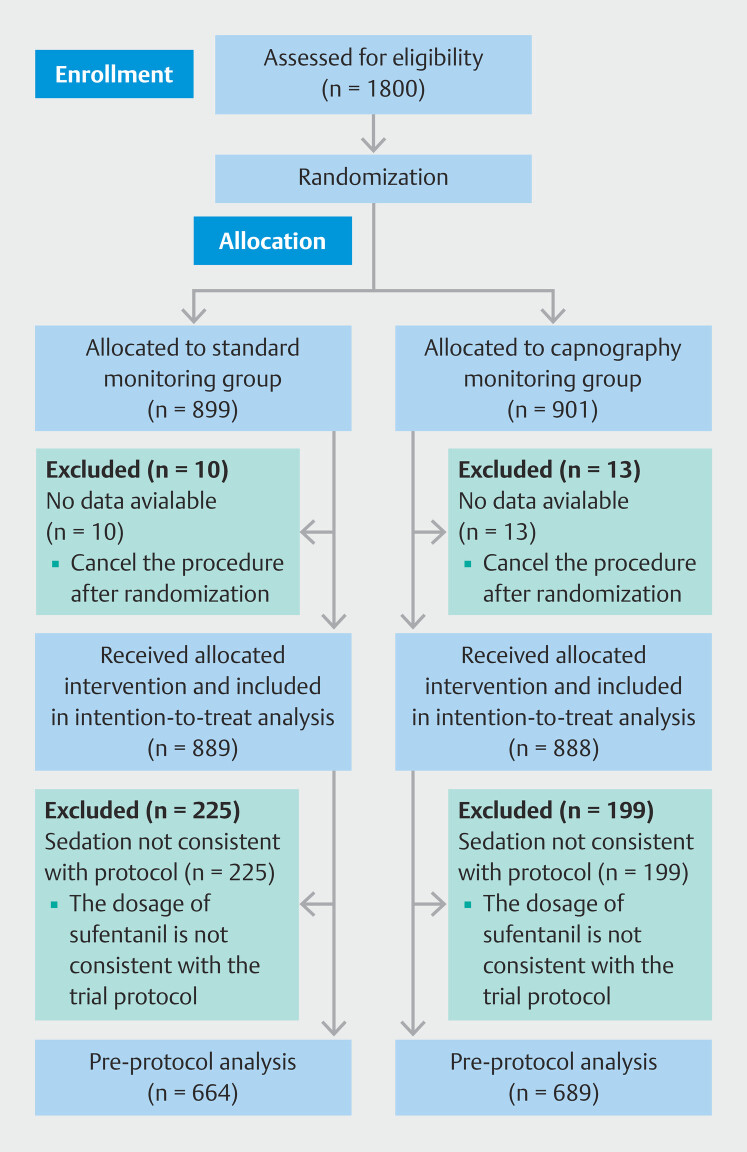
Experimental flow chart of the trial.

A total of 1,800 patients were enrolled. Among them, 13 in the intervention group and 10 in the control group were excluded from the intention-to-treat (ITT) analysis because the procedure was canceled after randomization. Thus, 1,777 patients were included in the ITT analysis. Of the 1,777 patients, 119 participants in the intervention group and 225 in the control group were excluded from the per-protocol set because the sedation dosage of sufentanil was inconsistent with the trial protocol ( Fig. 1 ).

### General characteristics

Table 1 shows participant baseline demographic, clinical, and procedural characteristics. Participant characteristics for both groups, including age, sex, body mass index (BMI), and ASA classification, were well matched at baseline. Airway-related variables, including Mallampati scores and snore, were comparable between the two groups.

**Table:** **Table 1** Demographic, clinical, and procedure characteristics of participants.

**Factor**	**Standard monitoring group (n = 889)**	**Capnography monitoring group (n = 888)**
Age [years, median (IQR)]	69 (66–72)	69 (66–72)
Male	422 (47)	456 (51)
Female	467 (53)	432 (49)
BMI [kg m ^2^ ; mead (SD)]	23 (3)	24 (3)
ASA physical status (1/2)	96 (11)/793 (89)	101 (11)/787 (89)
Procedure type (gastroscopy/colonoscopy/gastrointestinal endoscopy)	249 (26)/188 (21)/452 (51)	240 (27)/184 (21)/464 (52)
Initial propofol [mg, median (IQR)]	82 (70–100)	80 (70–100)
Initial sufentanil dose [ug, median (IQR)]	5 (5–7.5)	5 (5–7.5)
Mallampati classification (I/II/III/IV)	340 (38)/363 (41)/167 (19)/ 19 (2)	333 (37)/359 (41)/ 165 (19)/31 (3)
Snore	301 (34)	318 (36)
Baseline P _ET_ CO _2_ [kPa, median (IQR)]		35 (32–37)
Baseline SpO _2_ [%, median (IQR)]	99 (99–100)	99 (98–100)
ASA, American Society of Anesthesiologists; BMI, body mass index; IQR, interquartile range; P _ET_ CO _2_ , end-tidal carbon dioxide; SpO _2_ , pulse oxygen saturation.		

### Primary study outcome

**Fig. 2:**
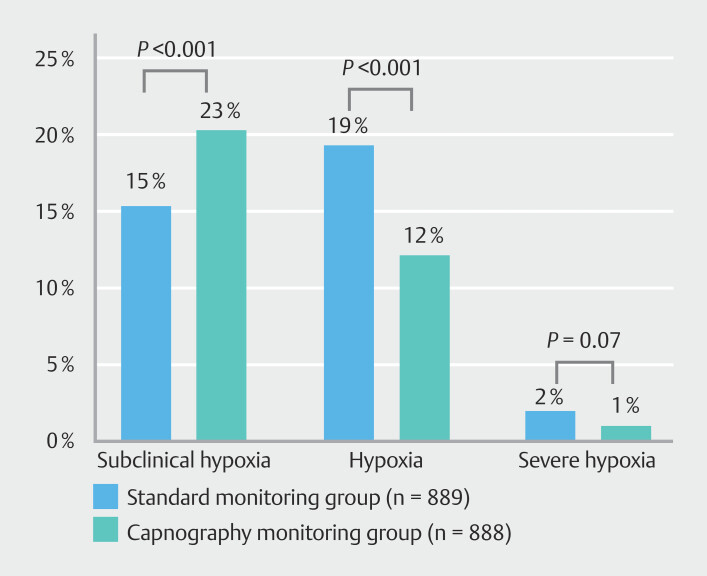
Rates of subclinical hypoxia, hypoxia, and severe hypoxia in two groups (Black column, standard monitoring group, n = 889; gray column, capnography monitoring group, n = 888.)

Capnography monitoring reduced incidence of hypoxia. Incidence of hypoxia was 12% (107/899) and 19% (171/901) in the intervention and control groups, respectively ( *P* < 0.001, Fig. 2 ). Statistical analysis results were maintained on per-protocol analysis. Incidence of hypoxia was 12% (81/689) and 19% (127/664) in the intervention and control groups, respectively ( *P* < 0.001, **Supplementary Table 1** ).

The intervention group showed a higher incidence of subclinical respiratory depression than the control group. Incidence of subclinical respiratory depression was 23% (202/899) and 15% (136/901) in the intervention and control groups, respectively ( *P* < 0.001 for difference, Fig. 2 ). Per-protocol analysis showed that incidence of subclinical respiratory depression was 23% (156/689) and 16% (104/664) in the intervention and control groups, respectively ( *P* = 0.002, **Supplementary Table 1** ).

Capnography monitoring did not reduce incidence of severe hypoxia. Incidence of severe hypoxia was 1% (8/899) and 2% (17/901) in the intervention and control groups, respectively ( *P* = 0.07, Fig. 2 ). Per-protocol analysis showed that incidence of severe hypoxia was 1% (7/689) and 2% (15/664) in the intervention and control groups, respectively ( *P* = 0.071, **Supplementary Table 1** ).

No significant differences in incidence of other AEs were observed between the intervention and control groups. No significant differences in incidence of sedation AEs were observed between the two groups. Based on capnography monitoring, 14.8% cases showed intraoperative ventilation changes, 67.9% cases showed apnea positive ( Table 2 ).

**Table:** **Table 2** Adverse events in two groups.

**Adverse events**	**Standard monitoring group (n = 899)**	**Capnography monitoring group (n = 888)**	***P* value **
Any adverse sedation event	29 (3.3)	36 (4.1)	0.374
Vomiting / Retching	1 (0.1)	0 (0)	0.500
Muscle rigidity, Myoclonus	0 (0)	2 (0.2)	0.479
Hypersalivation	0 (0)	1 (0.1)	0.500
Paradoxical response	0 (0)	1 (0.1)	0.500
Prolonged recovery	0 (0)	1 (0.1)	0.500
Failed sedation	0 (0)	2 (0.2)	0.479
Bradycardia	25 (2.8)	24 (2.7)	0.888
Hypotension	5 (0.6)	4 (0.5)	1.000
Ventricular arrhythmias	0 (0)	2 (0.2)	0.479
Tactile stimulation	1 (0.1)	1 (0.1)	1.000
Antisialogogue	0 (0)	1 (0.1)	0.500
Reversal agents	0 (0)	1 (0.1)	0.500
Atropine to treat bradycardia	9 (1.0)	9 (1.0)	0.998
Ephedrine to treat hypotension	5 (0.6)	4 (0.5)	1.000
Intraoperative ventilation change		131 (14.8)	
Apnea		603 (67.9)	

### Independent predictors of hypoxia on univariate and multiple regression analysis

Univariate and multiple regression analysis were performed with sex, age, BMI, ASA classification, procedure type, initial propofol dosage, Mallampati classification, and snore being independent variables to exclude a potential influence on the primary study outcome.

Univariate regression analysis revealed that female (1.4 (1.1, 1.8), P = 0.013), higher BMI (1.1 (1.1, 1.2), *P* < 0.001), performing gastrointestinal endoscopy at the same time (1.4 (1.1, 1.9), *P* = 0.019), higher Mallampati classification (1.5 (1.1, 2.0), P = 0.005), and snore (1.8 (1.4, 2.4), *P* < 0.001) were significant risk factors for hypoxia, whereas performing colonoscopy alone (0.4 (0.3, 0.7), *P* < 0.001) and high SpO2 before anesthesia (0.7 (0.7, 0.8), P < 0.001) were significant protective factors for hypoxia ( Table 3 ).

**Table:** **Table 3** Independent predictors of hypoxia on multivariable and univariable regression analysis in full analysis set.

**Variables**	**Univariable model**		**Multivariable model**	
	**OR (95%CI)**	***P* value **	**OR (95%CI)**	***P* value **
**Sex**				
Male	1 (ref)		1 (ref)	
Female	1.37 (1.07–1.76)	0.013	1.65 (1.25–2.18)	< 0.001
**Age**	1.03 (0.99–1.06)	0.16	1.04 (1.00–1.08)	0.050
** BMI (kg/m ^2^ ) **	1.14 (1.10–1.19)	< 0.001	1.12 (1.07–1.17)	< 0.001
**ASA physical status**				
1	1 (ref)		1 (ref)	
2	0.91 (0.62–1.34)	0.628	0.79 (0.51–1.22)	0.286
**Procedure type**				
Gastroscopy	1 (ref)		1 (ref)	
Colonoscopy	0.42 (0.27–0.67)	< 0.001	0.38 (0.24–0.61)	< 0.001
Gastrointestinal Endoscopy	1.41 (1.06–1.88)	0.019	1.41 (0.98–2.01)	0.063
**Initial propofol (mg)**	1.02 (1.01–1.02)	< 0.001	1.02 (1.01–1.02)	< 0.001
**Mallampati classification**				
1	1 (ref)		1 (ref)	
2	1.52 (1.13–2.03)	0.005	1.21 (0.88–1.67)	0.241
3	1.78 (1.26–2.51)	0.001	1.38 (0.93–2.05)	0.111
4	1.88 (0.93–3.80)	0.081	1.30 (0.59–2.89)	0.515
**Snore**				
No	1 (ref)		1 (ref)	
Yes	1.84 (1.44–2.37)	< 0.001	1.51 (1.13–2.02)	0.005
** Pre-anesthesia SpO _2_ (%) **	0.73 (0.67–0.80)	< 0.001	0.73 (0.66–0.81)	< 0.001
ASA, American Society of Anesthesiologists; BMI, body mass index; CI, confidence interval; OR, odds ratio; SpO _2_ , pulse oxygen saturation.				

Results of multiple regression analysis were consistent with those of univariate analysis ( Table 3 ). Per-protocol analysis set results of the univariate and multiple regression analysis were consistent with those of the full analysis set ( **Supplementary Table 2** ).

## Discussion

To the best of our knowledge, this is the first clinical trial to prove that capnography monitoring reduced incidence of hypoxia in gastrointestinal endoscopy under propofol sedation in older patients.

Older patients may suffer from multiple comorbidities 1 21 and be more likely to experience hypoxia during examination with sedative drugs because of their limited physiological reserves and reduced ventilatory responses to hypoxia and hypercapnia 21 . Therefore, it is of higher clinical significance to monitor PETCO2 than SpO _2_ in older patients.

A Colo Cap study showed that additional capnography monitoring of ventilatory activity reduces incidence of oxygen desaturation and hypoxia during propofol sedation for colonoscopy 17 . However, another study has proved that capnography monitoring did not improve safety or patient satisfaction, but did increase the cost of examination 20 . The current study demonstrated that capnography monitoring reduced incidence of hypoxia during sedated gastroscopy and colonoscopy in older patients.

Early interventions and treatment can be performed during the procedure based on changes in values and waveforms of capnography monitoring. The study showed that early intervention based on capnography monitoring significantly reduced incidence of hypoxia in older patients ( *P* < 0.001). Results of the clinical trial indicate that early interventions and treatment can be performed based on changes in values and waveforms of capnography monitoring to prevent hypoxia during IV anesthesia.

Incidence of severe hypoxia was lower in the intervention group than in the control group (1% vs. 2%). However, no statistically significant differences were observed ( *P* = 0.07). This might be because the primary study outcome was hypoxia, and the sample size was calculated based on incidence of hypoxia. If the sample size is large enough, incidence of severe hypoxia between the two groups may be statistically different, which needs to be proven in subsequent trials. In addition, capnography monitoring did not increase incidence of severe sedation-related AEs. All participants completed the endoscopy safely and did not undergo endotracheal intubation or laryngeal masks.

Univariate and multiple regression analyses showed that being female and having obesity were significant risk factors for hypoxia. Colonoscopy alone and high SpO _2_ before anesthesia were significant protective factors for hypoxia. Females are more sensitive than males to opioid receptor agonists. Females may experience respiratory depression and other adverse effects more easily if they are given the same doses as males 30 . Patients with obesity (BMI ≥ 28 kg/m ^2^ ) are characterized by poor lung-chest wall compliance, low lung capacity, small functional residual volume, and dysregulation of the alveolar ventilation flow ratio and are more prone to hypoxia in clinical anesthesia. During gastroscopy, a dental pad is placed in the patient's mouth 31 . In addition, the gastroscope and the gas released by the gastroscope (mostly carbon dioxide) affect patients’ respiratory rate and amplitude. This results in a higher incidence of hypoxia during gastroscopy than during colonoscopy. Besides, less time is required for colonoscopy than for gastroscopy and colonoscopy, and the amount of propofol used is less. Besides the influence of gastroscopy, patients undergoing gastroscopy and colonoscopy are more prone to hypoxia than those undergoing colonoscopy. The higher the SpO _2_ before anesthesia, the better the oxygen reserve and the more the tolerance to hypoxia. This can clarify why colonoscopy alone and high SpO _2_ before anesthesia were protective factors for development of hypoxia.

### Study limitations and future research

First, this was a single-blinded clinical study, which might cause potential bias. A double-blind study is required to determine generalizability of the findings. To avoid researcher bias, we formulated a strict and clear trial protocol and all treating physicians in our study were trained strictly. In addition, the selected patients were randomized and there is no other bias in the two groups. Second, the trial was not international, and the institutions participating in our experiment are all teaching hospitals in China. The medications or methods of sedation vary among different institutions and countries. Fortunately, the selected IV anesthetics are widely used, owing to their clinical compatibility, and the data obtained in this study could be represented in most clinical settings. Third, anesthesia professionals and not nurses administered the propofol, which leads to differences in our trial compared with the situation in other countries. Fourth, only older patients with ASA grades I–II were included in this trial, and those with ASA grades III–IV were excluded. Fifth, in our study, we lack the patient-centered evaluation indicators.

Patient-centeredness is considered a quality indicator in gastrointestinal endoscopy 32 . Sixth, the study lacks information about cost. Analyzing the increase in cost of additional monitoring versus the actual benefit could have made the trial more meaningful. Thus, further studies are needed that include older patients with higher ASA classifications.

## Conclusions

Early intervention based on capnography monitoring of hypoventilation reduces incidence of hypoxia in older patients undergoing gastrointestinal endoscopy under propofol sedation.

## Footnotes

1 Shared last authorship

## Supplementary Material

Supplementary Material Zusatzmaterial
