# Supplementary material for: Capnography monitoring reduces incidence of hypoxia in older patients undergoing gastrointestinal endoscopy under propofol sedation
Source: Endosc Int Open. 2025 Aug 7;13:a26636372. doi: 10.1055/a-2663-6372 (PMC12371663; doi:10.1055/a-2663-6372)
Supplement: Supplementary file 1 — Supplementary Material [file 10-1055-a-2663-6372_26707926.pdf]

Supplementary Table 1 Primary and secondary study outcomes in the two groups in per-protocol analysis set.

**Factor**	**Standard monitoring group (n = 899)**	**Capnography monitoring group (n = 888)**	***P* value**
**Primary outcome**			
Hypoxia	127 (19)	81 (12)	< 0.001
**Secondary outcomes**			
Subclinical hypoxia	104 (16)	156 (23)	0.002
Severe hypoxia	15 (2)	7 (1)	0.071

Endosc Int Open | DOI 10.1055/a-2663-6372 | © The Author(s).

Supplementary Table 2 Independent predictors of hypoxia on multivariable and univariable regression analysis in per-protocol analysis set.

Variables	Univariable model		Multivariable model	
	**OR (95%CI)**	***P* value**	**OR (95%CI)**	***P* value**
**Sex**				
male	1 (ref)		1 (ref)	
female	1.34 (1.01-1.79)	0.046	2.50 (1.76-3.54)	< 0.001
**Age**	1.04 (1.00-1.08)	0.075	1.08 (1.03-1.13)	0.001
**BMI (kg/m^2)**	1.15 (1.09-1.20)	< 0.001	1.04 (0.98-1.10)	0.172
**ASA physical status**				
1	1 (ref)		1 (ref)	
2	1.34 (0.82-2.17)	0.239	1.54 (0. 89-2.64)	0.122
**Procedure type**				
Gastroscopy	1 (ref)		1(ref)	
Colonoscopy	0.37 (0.23-0.59)	< 0.001	0.38 (0.23-0.63)	< 0.001
Gastrointestinal Endoscopy	1.59 (1.16-2.18)	0.004	2.01 (1.41-2.87)	< 0.001
**Initial propofol (mg)**	1.02 (1.01-1.03)	< 0.001	1.03 (1.02-1.04)	< 0.001
**Mallampati classification**				
1	1 (ref)		1 (ref)	
2	1.65 (1.18-2.31)	0.003	1.32 (0.91-1.93)	0.148
3	1.94 (1.31-2.87)	0.001	1.65 (1.05-2.59)	0.031
4	1.68 (0.78-3.63)	0.185	1.85 (0.34-2.13)	0.726
**Snore**				
No	1 (ref)		1 (ref)	
Yes	2.04 (1.53-2.71)	< 0.001	1.53 (1.09-2.14)	0.015
**Pre-anesthesia SpO_2 (%)**	0.75 (0.68-0.84)	< 0.001	0.75 (0.66-0.84)	< 0.001

ASA, American Society of Anesthesiologists; BMI, body mass index; CI, confidence interval; OR, odds ratio; SpO_2, pulse oxygen saturation.
